# Long term outcome after combined modality treatment for anal cancer

**DOI:** 10.2478/v10019-012-0022-2

**Published:** 2012-04-11

**Authors:** Irena Oblak, Primoz Petric, Franc Anderluh, Vaneja Velenik, Peter Albert Fras

**Affiliations:** Department of Radiotherapy, Institute of Oncology Ljubljana, Ljubljana, Slovenia

**Keywords:** anal cancer, radiochemotherapy, salvage surgery, outcome

## Abstract

**Background:**

The aim of the retrospective study was to evaluate the effectiveness and toxicity of radiochemotherapy in patients with squamous cell carcinoma of the anal canal treated at a single institution.

**Patients and methods:**

Between 1/2003 and 9/2010, 84 patients were treated with radical radiochemotherapy at the Institute of Oncology Ljubljana, Slovenia. The treatment consisted of 3-dimensional conformal external beam radiotherapy with concurrent chemotherapy (5-fluorouracil and mytomycin C), followed by brachytherapy or external beam boost. The toxicity of therapy and its effectiveness were assessed.

**Results:**

The treatment was completed according to the protocol in 79.8% of patients. The median follow-up time of 55 survivors was 53 months (range: 16–105 months). The 5-year locoregional control (LRC), disease-free survival (DFS), disease-specific survival (DSS), overall survival (OS) and colostomy-free survival (CFS) rates were 71%, 68%, 81%, 67% and 85%, respectively. No treatment-related mortality was observed. The most frequent acute side-effect of the treatment was radiodermatitis (grade 3–4 in 58.2% of patients). LENT-SOMA grade 3–4 late radiation side effects were observed in 15 (18%) patients. In patients with brachytherapy boost a trend of less late side effects was observed compared to patients with external beam boost (P=0.066). On multivariate analysis, complete clinical disease response was identified as an independent prognostic factor for LRC, DFS and DSS, the salvage surgery for LRC and DFS, whereas Hb below 120 g/l retained its independent prognostic value for OS.

**Conclusions:**

Radiochemotherapy provides an excellent disease control and the survival with preserving anal sphincter function in majority of patients. Surgical salvage with abdominoperineal resection for persistent or recurrent disease has curative potential.

## Introduction

Anal cancer is a relatively rare tumour, representing 2–4% of all cancers of colorectum and anus.^1^ Women are more often affected than men. During the past decades, the incidence in developing countries has increased, mostly in young homosexual men, perhaps due to sexual transmission of human papilloma virus (HPV) and human immunodeficiency virus (HIV), which are known causal factors in this and other cancers.^1^,^2^ Anal cancer is predominantly a locoregional disease and distant metastases are found in 5–10% of the patients.^1^,^3^–^8^ Following publications on organ preserving treatment in the eighties^9^, the treatment paradigm has shifted from abdomino-perineal resection (APE) with permanent colostomy to radical radiochemotherapy, resulting in sphincter preservation rates of around 80%, even in cases with locally advanced disease. Surgery is indicated only in cases of residual or recurrent tumour and for complications of radiotherapy.^1^,^10^

Numerous trials have demonstrated complete response rates of 80–90%, with high local control, survival and sphincter preservation rates following radical radiochemotherapy.^3^,^10^–^12^ The purpose of our retrospective study was to evaluate the effectiveness and toxicity of radiochemotherapy in a single-centre prospective cohort of patients with squamous cell carcinoma of the anal canal.

## Patients and methods

### Patients and tumour characteristics

Eighty-six patients with biopsy proven cancer of the anal canal were treated at the Institute of Oncology Ljubljana between January 2003 and September 2010. Two patients with distant metastases were treated with palliative intent and were excluded from present analysis. The remaining 84 patients (48 females and 36 males) were treated with curative intent. Mean age was 63 years (range: 34–87 years). According to the UICC TNM staging criteria, 6 (7.1%) patients had stage I, 48 (57.1%) stage II, 14 (16.7%) stage IIIA and 16 (19%) stage IIIB disease.^13^

### Investigations before and during therapy

The multidisciplinary approach is the policy of treatment for all cancer patients at the Institute of Oncology Ljubljana^14^; therefore, all patients were presented to a multidisciplinary advisory team, consisting of a surgeon, radiation oncologist and medical oncologist, in order to assess the prospects of the treatment. All patients underwent a general clinical examination, blood tests, chest radiography and abdominopelvic computed tomography (CT). Locoregional extent of the disease was evaluated with anorectal examination (performed by a surgeon and a radiation oncologist), rectoscopy, endoscopic ultrasound (US) and magnetic resonance imaging (MRI) of the pelvis. In cases, suspicious for inguinal lymph node involvement, fine needle aspiration biopsy was performed. Detailed pre-treatment clinical drawings and photographs were taken and tumour borders tattooed on peri-anal skin for the purpose of brachytherapy (BT) treatment planning.

During the treatment, weekly clinical examination and blood tests were performed. The acute treatment related toxicity was assessed according to National Cancer Institute Common Toxicity Criteria (NCI-CTC) version 2.0.^15^

### Treatment

A planned treatment schedule consisted of 3-dimensional (3D) conformal external beam radiotherapy (EBRT) with concurrent chemotherapy (ChT), followed by brachytherapy (BT) or EBRT boost. 3D conformal EBRT was delivered using a four-field box technique at a 15 MV linear accelerator. Clinical target volume (CTV) included the gross tumour volume with a safety margin of 1.5–2 cm in all directions and the regional lymph node areas. To arrive at a planning target volume (PTV), an additional margin of 0.7 cm in all directions was applied to the CTV. A nominal dose of 45 Gy (1.8 Gy daily fractions, five fractions a week) was prescribed to ≥95% of the PTV. Prophylactic bilateral inguinal EBRT was given to 45–50 Gy by anterior photon beam and adequate additional electron beam complements of adequate energy to reach the deepest portion of these nodes. In cases with inguinal lymph node metastases, the involved areas were irradiated with separate electron fields to a total dose of 60 Gy.

Concurrent ChT was planned in all but stage I patients and patients with significant medical comorbidities. ChT consisted of two cycles of 5-fluorouracil (5-FU) (daily dose of 1000 mg/m^2^ in 96-hours continuous infusion), given during weeks 1 and 5 of EBRT. Mitomycin C (10 mg/m^2^ in bolus intravenous injection) was administered on day 1 of the first ChT cycle.

After delivery of 45 Gy of EBRT+/-ChT, a boost dose was planned. In tumours, larger than 5 cm or N2-3 disease, the boost was applied with EBRT, whereas in all other cases an interstitial pulsed-dose rate BT boost was delivered. CTV at the time of BT corresponded to initial tumour extension, as documented by pre-treatment clinical drawings, imaging examinations, photographs and tattooed markings of tumour borders on perianal skin. Metal needles were implanted through a perineal template homogeneously in the CTV, respecting the rules of the Paris system. The distance between needles and ano-rectal mucosa was kept above 5 mm. This was assured by palpation during the needle insertion and by transrectal US. The anal cylinder was inserted to displace uninvolved ano-rectal mucosa from the high dose region. Until 2006, treatment planning was based on two orthogonal radiographs. From then on, CT based treatment planning was introduced. A biologically equivalent dose of 15–30 Gy was prescribed to the reference isodose line, corresponding to the 85% of mean basal dose (linear quadratic model, assuming an α/β of 10 Gy and 3 Gy for the tumour and late reacting normal tissues, respectively, sublethal damage repair half time of 1.5 hours, reference dose rate of 0.5 Gy per hour). The prescribed dose was chosen depending on initial tumour burden and extent of regression during EBRT. After introduction of CT into treatment planning, subtle individualized 3D-optimization of dose distribution was performed to increase the dose to the CTV while respecting the normal tissues tolerance.

In cases of severe treatment-related toxicity, irradiation and/or ChT doses were modified and adapted to the patient’s physical condition or laboratory findings. When necessary, ChT application was delayed, or EBRT was temporarily interrupted or terminated.

### Follow-up after treatment

The first post-treatment follow-up visit was performed by a senior radiation oncologist 6 weeks after the completion of radiotherapy. A response to the treatment was evaluated by clinical examination, appropriate imaging studies (MRI, US) and biopsies, when indicated. In patients with clinical complete remission, follow-up investigations were carried out at 3 month-intervals thereafter. In cases of the incomplete response, the clinical evaluation was repeated every 6 weeks until the complete remission was recorded. In cases with evidence of progression or recurrence after the end of the treatment, surgery (APE) was recommended.

Late normal tissue side effects (events occurring 3 months or more after the end of the treatment) were assessed at time of each follow-up evaluation, employing the LENT-SOMA scoring system.^16^

### Statistical analysis and ethical consideration

A statistical analysis was performed using personal computer and software statistical package SPSS, version 18 (SPSS Inc., USA).

The main endpoints of the study were as follows: response to therapy, locoregional control (LRC, the event was local or regional recurrence), disease-free survival (DFS, the event was local, regional or systemic recurrence), disease-specific survival (DSS, the event was death due to the carcinoma of the anal canal), overall survival (OS, the event was death from any cause) and colostomy-free survival (CFS, the event is need for colostomy).

The survival of patients was computed from the date of the treatment start to December 1, 2011 (close-out date). Survival probability was calculated using Kaplan-Meier estimate, and log-rank test was used to evaluate the influence of individual prognostic factors (age, performance status, T-, N-and overall stage, radiotherapy and chemotherapy dose), on the analysed endpoints.^17^,^18^ Independent prognostic values of the factors that appeared statistically significant on the univariate analysis, were tested by the multivariate Cox regression analysis model.^19^ Two-sided tests were used and the differences at P<0.05 were considered as statistically significant.

The retrospective study was carried out according to the Declaration of Helsinki.

## Results

### Course of treatment

Median duration of EBRT and total treatment time was 36 days (range: 29–72 days) and 57 days (range: 30–98 days), respectively. Sixty-seven (79.8%) patients completed the treatment according to the protocol. Total EBRT dose of 45 Gy was applied in 82 (97.6%) patients. In two, due to acute side effects, EBRT was stopped at 18 and 25 Gy, respectively, but the treatment was completed with BT (TD: 30–35 Gy).

During EBRT, two cycles of 5-FU were administered as planned in 67 (79.8%) patients. Eight (14%) patients received one cycle only due to adverse side effects during the treatment. Concomitant capecitabine was administered in one patient who was primarily operated for locoregionally advanced colon carcinoma and in who during preoperative investigations synchronous anal carcinoma was found. Chemotherapy was omitted in 6 (7.1%) and 3 (3.6%) patients due to stage I disease and severe comorbidity, respectively.

Boost with the median dose of 14.4 Gy (range: 6–20 Gy) to the primary tumour was applied through reduced photon fields in 33 (39.3%) patients. Interstitial BT boost was performed in 49 (58.3%) patients after EBRT with a mean interval of 27 days (range: 6–57 days). Boost was omitted in two (2.4%) patients because of treatment side effects in one case and the other patient refused the further treatment.

### Acute side effects

The treatment was well tolerated in the majority of patients and no treatment-related mortality was observed. Frequency and intensity of acute adverse side effects are listed in Table 1. The most frequent grade 3 side-effect was radiodermatitis, occurring in 48 (57%) patients during EBRT. One patient developed grade 4 radiodermatitis. All cases of radiodermatitis healed without consequences.

### Outcome

Median follow-up time was 43 months (range: 8–105 months) in all patients and 53 months (range: 16–105 months) in survivors.

At the first follow-up evaluation, which was done 6 weeks after the treatment, the complete clinical remission of the tumour was found in 55 (65.5%) patients. At 18 weeks after the end of the treatment, complete remission, partial response and stable disease were recorded in 67 (79.8%), 12 (14.3%) and 4 (4.8%) patients, respectively. In one patient, there was evidence of tumour progression during the treatment.

Five patients with complete response later developed local or locoregional recurrence after a median period of 8 months (range: 4–26 months). In two patients with complete response, distant metastases without local recurrence occurred.

Of 24 (28.6%) patients with persistent disease or locoregional recurrence, 12 (14.3%) were treated surgically. In 11 patients, APE was performed and one patient had inguinal dissection due to a recurrence in inguinal lymph nodes. Other 12 (14.3%) patients had unresectable disease. Three of the operated patients died of the recurrent disease, others are alive without evidence of the disease.

On the study close-out date, 55 (65.5%) patients were alive, 51 (92.7%) of them being disease free. Fifteen (17.9%) patients died from the anal canal cancer. One (1.2%) patient, who experienced locoregional recurrence, died from metachronous bronchus cancer, four (4.8%) patients died from metastatic breast cancer, metastatic colon cancer, metastatic bronchus cancer and metastatic malignant melanoma, six patients (7.1%) died from vascular events and in three (3.5%) patients the cause of death could not be determined.

**FIGURE 1:**
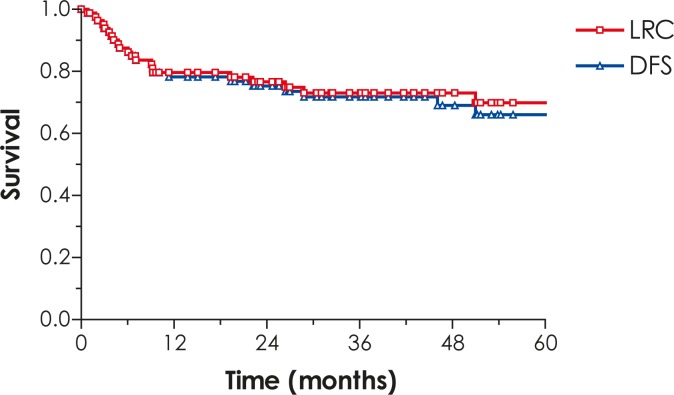
Locoregional control (LRC) and disease-free survival (DFS).

**FIGURE 2:**
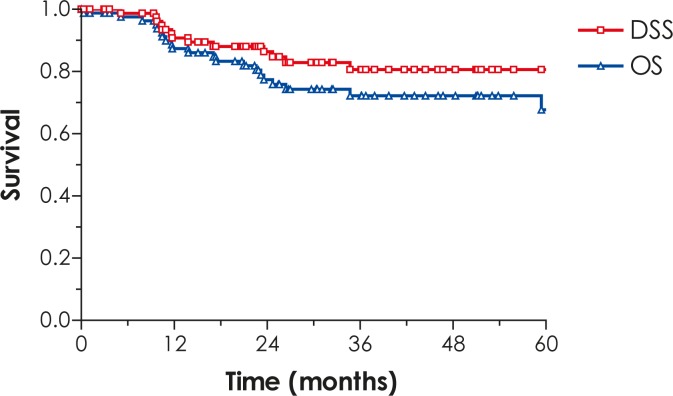
Disease-specific survival (DSS) and overall survival (OS).

**FIGURE 3:**
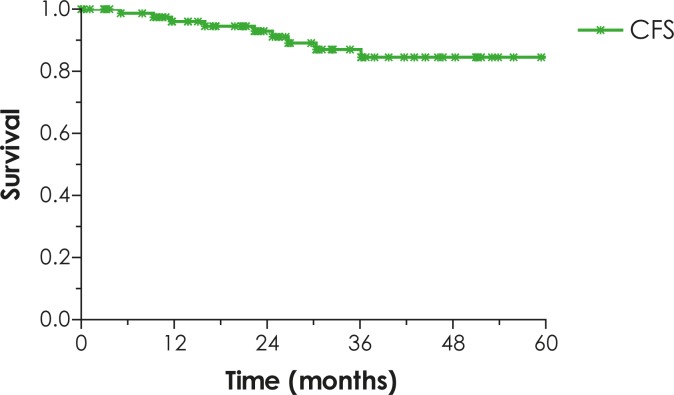
Colostomy-free survival (CFS).

The 5-year LRC, DFS, DSS, OS and CFS rates for all patients are 71%, 68%, 81%, 67% and 85%, respectively (Figure 1–3).

### Late side effects

Grade 3–4 late radiation side effects according to the LENT-SOMA scoring system^13^ were observed in 15 (18%) patients. Three (4%) patients experienced post-treatment anal stenosis, requiring repeated dilatations and two (2%) developed chronic non-healing ulcer at the anal verge. Nine (11%) patients had grade 3 incontinence of anal sphincter. In one patient without disease recurrence, colostomy was performed due to severe anal sphincter disfunction. In one patient with anal stenosis, hematuria was observed, as well. Forty-nine (58.3%) patients with BT boost on primary tumour had less late site effects compared to 33 (39.3%) patients with EBRT boost, but the difference was not significant (P=0.066).

### Prognostic Factors

On the univariate analysis, patients with locally advanced disease (T3-4) and incomplete response had worse LRC and all studied survival endpoints when compared to their counterparts.

Patients with the involvement of lymph nodes and patients with overall disease stage III had worse LRC, DFS, DSS and CFS in comparison with patients with N0 and overall stage I or II and patients with Hb below 120 g/l had worse LRC, DFS, DSS and OS in comparison with patients with Hb 120 g/l or higher. In addition, patients with poor performance status (WHO 1 or 2) had worse OS and patients with overall treatment time over 73 days had worse LRC. The patients with salvage surgery (APE or nodal dissection) for residual disease or tumour and/or regional lymph node recurrence had worse LRC, DFS and CFS, but not DSS and OS compared to complete responders.

For other analysed factors (sex, age, treatment intensity and the method of radiotherapy boost) no impact on the outcome was found.

On the multivariate analysis, a complete clinical disease response was identified as an independent prognostic factor for LRC, DFS and DSS, the salvage surgery for LRC and DFS, whereas Hb below 120 g/l retained its independent prognostic value for OS, and for LRC it was on the threshold of statistical significance (P=0.061) (Table 2).

## Discussion

Before Negro *et al.* in 1974 reported that a complete tumour response can be achieved with radiochemotherapy, APE was the standard of the treatment in patients with anal cancer.^9^ Nowadays, radiotherapy with concomitant ChT represents a standard treatment of anal cancer. Complete response rates and 5-year OS in patients with early stage disease range from 80–90% and 95–100%, respectively, and in patients with tumours larger than 5 cm from 50–75% and 35–70%.^1^,^3^,^11^,^20^ In our study the complete response was recorded in 67 (79.8%) patients, regardless of the stage. Results of our analysis compare favourably to other published studies.^1^,^3^,^19^,^20^–^22^ According to the data of the Cancer Registry of Slovenia, 24 (24%) patients were not referred to the treatment with radiotherapy in the period between 2003 and 2007.^4^–^8^ We can only speculate that these patients were treated with local excision and were not presented to multidisciplinary advisory board. It could be debated if all these patients had an appropriate treatment, since it is well known that the local excision should be reserved only for small, well differentiated mucosal or submucosal tumours (<2 cm) and without sphincter involvement.^1^

Although in 24 (28.6%) patients the complete clinical response could not be achieved or they had recurrent disease, in only 12 (50%) patients salvage surgery was possible and only 8 (66.7%) of these operated patients were free of the disease. APE was performed in 11 patients and in one patient bilateral nodal dissection was carried out due to a solitary lymph node involvement. In one patient, APE was necessary due to severe sphincter incontinence after the end of the treatment. Our results on the salvage surgery rate are comparable to results of Ajani *et al*. and Peiffert *et al.* with salvage APE rate of 16% and 10%, respectively.^22^,^23^ In the study of Akbari *et al.*, where salvage surgery was performed in 57 patients with persistent or recurrent disease, the 5-year OS for all patients was 33%, whereas in our study it was 67%.^24^ As the median follow-up time in Akbari’s study was 34.1 months, whereas in ours it was 43 months (range: 8–105 months), the direct comparison of reported results of these two studies could be misleading. However, the observed 75% rate of disease-free patients after salvage surgery is without doubt encouraging.

It is well known that patients with a complete tumour response following radiochemotherapy have a better local control and survival, which was demonstrated in our series, as well.^3^,^23^,^25^ We found out that a complete clinical disease response was an independent prognostic factor for LRC, DFS and DSS.

The patients who had salvage surgery had worse LRC and DFS but it is encouraging that no statistically significant difference in DSS and OS was found. We can conclude that patients in whom salvage surgery was performed had similar OS as patients with the complete tumour remission. In some, but not all series, they reported that residual or recurrent carcinoma of anal canal after radiochemotherapy was associated with poor outcome after the attempted salvage surgery.^25^ Furthermore, they found out that APE is successful as salvage therapy in about 50% of patients with local disease only but salvage rate is very poor in patients that have nodal involvement or residual or recurrent carcinoma which is fixed to the pelvic sidewall.^26^–^27^

In our study Hb below 120 g/l was identified as an independent prognostic factor for OS. It is not surprising, because anaemia is namely a well known prognostic factor for the lower tumour control and the survival in patients who are treated with radiotherapy.^28^ It may be related with hypoxia and consequent development of tumour cells’ radioresistency.^29^

At first evaluation at 6 weeks after the end of the treatment, the complete tumour remission was found in only 55 (65.5%) of patients, while another 12 (14.3%) patients reached the complete remission at 18 weeks post treatment. There are several other reports of very slow disease regression, with a complete response observed even up to 6–9 months after the treatment was completed.^1^,^30^,^31^ The evaluation recommendations suggest that if there is no progression of the disease, a careful »wait and see« policy with repeated biopsies may be advocated. However, in cases of persistent disease or tumour progression, APE is recommended following the histological confirmation of the presence of viable malignant cells.

In our study, the profile and frequency of acute and late treatment-related toxic side effects were comparable to reports of other researchers.^2^,^10^,^32^ The most frequently reported acute side effect was radiodermatitis with grade 3 or 4 occurring in 58% of patients during EBRT, whereas 15 (18%) patients experienced grade 3 or 4 late radiation side effects. Three (4%) patients experienced post-treatment anal stenosis, two (2%) developed chronic non-healing ulcer at the anal verge, 9 (11%) patients had grade 3 incontinence of anal sphincter and in one patient without disease recurrence, colostomy was performed due to the severe anal sphincter dysfunction. The rate of our late side effects is similar to other reports.^3^,^11^,^22^,^32^,^33^

The patients with BT boost on primary tumour had less late side effects (P=0.066). We should emphasize that these patients had less advanced tumours and, correspondingly, smaller tissue volumes were irradiated during boost phase of the treatment. Furthermore, it is well known that patients with BT boost have less late toxicity when compared to EBRT boost, because the use of interstitial implant has the advantage of more focused escalation of irradiation dose, resulting in more efficient sparing of the surrounding normal tissues.^34^

There are several possibilities for the improvement in the disease control and the survival in the future. In locally advanced disease, innovative approaches with 3D image-based BT boost and intensity modulated radiotherapy offer a potential for the individualised escalation of the target dose while respecting normal tissue tolerance.^35^ For the treatment of unresectable recurrences and distant metastases, the development of more active new anti-cancer drugs, for example epidermal growth factor receptor (EGFR) inhibitors may represent an option. Finally, the majority of the anal cancers are causally connected to the persistent HPV infection, so it can be assumed that the HPV vaccines may become an important prevention measure against anal cancer in the future.

To conclude, radiochemotherapy provides an excellent disease control and the survival with preserving anal sphincter function in majority of patients which were evidenced also by our results. Surgical salvage with APE for persistent or recurrent disease should be considered whenever applicable as it can be curative in substantial proportion of such patients.

## Figures and Tables

**TABLE 1:** Acute treatment toxicities

**Toxicity**	**NCI**^12^ **grade (%)**					
	**0**	**1**	**2**	**3**	**4**	**Total**
Stomatitis	61	19	12	8	0	100
Nausea, vomiting	82	10	6	2	0	100
Diarrhoea	61	18	11	9	1	100
Radiodermatitis	12	14	16	57	1	100
Infection	55	17	18	7	3	100
Leucocyte count	43	31	18	7	1	100
Haemoglobin level	46	46	7	0	1	100
Platelet count	69	27	3	1	0	100

**TABLE 2:** Univariate and multivariate analysis of survival

**Prognostic factor**	**N**	**Locoregional control**			**Disease free survival**			**Disease specific survival**			**Overall survival**			**Colostomy free survival**		

		**UVA**		**MVA**	**UVA**		**MVA**	**UVA**		**MVA**	**UVA**		**MVA**	**UVA**		**MVA**

		**%**	**p-value**	**p-value**	**%**	**p-value**	**p-value**	**%**	**p-value**	**p-value**	**%**	**p-value**	**p-value**	**%**	**p-value**	**p-value**
T-stage																
T1+2	45	80.3			80.3			92.3			75			94.4		
T3+4	39	55	0.016		46.6	0.004		65.2	0.009		57.5	0.049		45.9	0.01	
N-stage																
N0	56	78.6			75			90.8			73.7			89.8		
N+	28	50.6	0.028		47.8	0.018		59.4	0.002		56.9	0.059		71.7	0.015	
Overall stage																
Stage I+II	54	78.5			74.9			90.8			73.6			89.8		
Stage III	30	50.8	0.031		48	0.02		59.4	0.002		56.9	0.061		71.7	0.015	
PS																
0	58	75.2			75.2			81.3			77.9			84.4		
1+2	26	54,5			43.4			78.9			39.4	0.041		84.7		
OTT																
<73 days	70	73.4			69			83			72.3			70.6		
≥ 73 days	14	56.3	0.043		56.3	0.062		67.5			44.2	0.062		60.9	0.086	
CR																
Yes	67	91.1			85.6			95.9			80			66.8		
No	17	5.9	<0.001	<0.001	5.9	<0.001	<0.001	22.4	<0.001	<0.001	21	<0.001		59.3	0.014	
Hb																
< 120 g/l	47	58.5			52.6			68			45.5			78.7		
≥ 120 g/l	37	82.7	0.016	0.061	80	0.017		94	0.015		90.7	0.001	0.007	89.6		
SS																
Yes	13	30.8			30.8			71.9			67.3			10.1		
No	71	77.9	<0.001	0.01	73.3	<0.001	0.003	82.4			71.9			100	<0.001	

N, number of patients; UVA, univariate analysis; MVA, multivariate analysis; PS, performance status; OTT, overall treatment time; CR, complete response; Hb, hemoglobin; SS, salvage surgery.

## References

[1] Rousseau DL, Thomas CR, Petrelli NJ, Kahlenberg MS (2005). Sqamous cell carcinoma of the anal canal. Surg Oncol.

[2] But-Hadzic J, Jenko K, Poljak M, Kocjan BJ, Gale N, Strojan P (2011). Sinonasal inverted papilloma associated with squamous cell carcinoma. Radiol Oncol.

[3] Chapet O, Gerard JP, Riche B, Alessio A, Mornex F, Romestaing P (2005). Prognostic value of tumour regression evaluated after first course of radiotherapy for anal canal cancer. Int J Radiat Oncol Biol Phys.

[4] (2006). Cancer incidence in Slovenia 2003.

[5] (2007). Cancer incidence in Slovenia 2004.

[6] (2008). Cancer incidence in Slovenia 2005.

[7] (2009). Cancer incidence in Slovenia 2006.

[8] (2010). Cancer incidence in Slovenia 2007.

[9] Nigro ND, Vaitkevicius VK, Considine D (1974). Combined therapy for cancer of the anal canal. Dis Colon Rectum.

[10] Fraunholz I, Rebeneck D, Weiß C, Rödel C (2010). Combined-modality treatment for anal cancer. Strahlenther Oncol.

[11] Ferrigno R, Nakamura RA, Dos Santos Novaes PE, Pellizzon AC, Maia MA, Fogarolli RC (2005). Radiochemotherapy in the conservative treatment of anal canal carcinoma: Retrospective analysis of the results and radiation dose effectiveness. Int J Radiat Oncol Biol Phys.

[12] Oblak I, Petric P, Anderluh F, Velenik V, Hudej R, Fras AP (2009). Anal cancer chemoirradiation with curative intent - a single institution experience. Neoplasma.

[13] Sobin LH, Gospodarowitcz MK, Wittekind C (2009). International Union Against Cancer (UICC): TNM classification of malignant tumours.

[14] Strojan P (2010). Role of radiotherapy in melanoma management. Radiol Oncol.

[15] Ajani JA, Welch SR, Raber MN, Fiels WS, Krakoff IM (1990). Comprehensive criteria for assessing therapy-induced toxicity. Cancer Invest.

[16] (1995). LENT-SOMA scales for all anatomic sites. Int J Radiat Oncol Biol Phys.

[17] Kaplan EL, Meier P (1958). Nonparametric estimation from incomplete observations. J Am Stat Assoc.

[18] Peto R, Pike MC, Armitage P, Breslow NE, Cox DR, Howard SV (1977). Design and analysis of randomized clinical trials requiring prolonged observation of each patient. II. Analysis and examples. Br J Cancer.

[19] Cox DR (1972). Regression models and life-tables. J R Stat Soc Bull.

[20] Gerard JP, Ayzac L, Hun D, Romestaing P, Coquard R, Ardiet JM (1998). Treatment of anal canal carcinoma with high dose radiation therapy and concomitant fluorouracil-cisplatinum. Long-term results in 95 patients. Radiother Oncol.

[21] Flam M, John M, Pajak TF, Petrelli N, Myerson R, Doggett S (1996). Role of mitomycin in combination with florouracil and radiotherapy, and a salvage chemoradiotherapy in the definitive nonsurgical treatment of the epidermoid carcinoma of the anal canal: results of a phase III randomozed intergroup study. J Clin Oncol.

[22] Ajani JA, Winter KA, Gunderson LL, Pedersen J, Benson AB, Thomas CR (2008). Fluorouracil, mitomycin, and radiotherapy vs fluorouracil, cisplatin, and radiotherapy for carcinoma of the anal canal: a randomized controlled trial. JAMA.

[23] Peiffert D, Bey P, Pernot M, Guillemin F, Luporsi E, Hoffstetter S (1997). Conservative treatment by irradiation of epidermoid cancers of the anal canal. Prognostic factors of tumoural control and complications. Int J Radiat Oncol Biol Phys.

[24] Akbari RP, Paty PB, Guillem JG, Weiser MR, Temple LK, Minsky BD (2004). Oncologic outcomes of salvage surgery for epidermoid carcinoma of the anus initially managed with combined modality therapy. Dis Colon Rectum.

[25] Cummings BJ, Brierley JD, Halperin EC, Perez CA, Brady LW (2008). Anal Cancer. Principles and Practice of Radiation Oncology.

[26] Schiller DE, Cummings BJ, Rai S, Le LW, Last L, Davey P (2007). Outcomes of salvage surgery for squamous cell carcinoma of the anal canal. Ann Surg Oncol.

[27] Pocard M, Tiret E, Nugent K, Dehni N, Parc R (1998). Results of salvage abdominoperineal resection for anal cancer after radiotherapy. Dis Colon Rectum.

[28] Oblak I, Strojan P, Zakotnik B, Budihna M, Smid L (2003). Hemoglobin as a factor influencing the outcome in inoperable oropharyngeal carcinoma treated by concomitant radiochemotherapy. Neoplasma.

[29] Horsman MR, Van der Kogel A, Joiner M, Van der Kogel A (2009). Therapeutic approaches to tumour hypoxia. Basic Clinical Radiobiology.

[30] Sato H, Koh PK, Bartolo DCC (2005). Management of anal canal cancer. Dis Colon Rectum.

[31] Cummings BJ, Keane TJ, O’Sullivan B, Wong CS, Catton CN (1991). Epidermoid anal cancer: treatment by radiation alone or by radiation and 5-fluorouracil with and without mitomycin C. Int J Radiat Oncol Biol Phys.

[32] Marshall DT, Thomas CR (2009). Carcinoma of the anal canal. Oncol Rev.

[33] Mitchell SE, Mendenhal WM, Zlotecki RA, Carroll RR (2001). Squamous cell carcinoma of the anal canal. Int J Radiat Oncol Biol Phys.

[34] Hwang JM, Rao AR, Cosmatos HA, Wang R, Kaptein JS, Kagan RA (2004). Treatment of T3 and T4 anal carcinoma with combined chemotharapy and interstitial 192-Ir implantation: a 10-year experience. Brachytherapy.

[35] Bailey DW, Kumaraswamy L, Podgorsak MB (2010). A fully electronic intensity-modulated radiation therapy quality assurance (IMRT QA) process implemented in a network comprised of independent treatment planning, record and verify, and delivery systems. Radiol Oncol.

